# Appropriateness of digoxin measurement in hospitalized patients

**DOI:** 10.11613/BM.2018.010901

**Published:** 2017-11-24

**Authors:** Seyma Oncu, Ayse Gelal, Ozgur Aslan, Reyhan S Ucku

**Affiliations:** 1 Department of Pharmacology, Dokuz Eylul University Medical Faculty, Izmir, Turkey; 2 Department of Cardiology, Dokuz Eylul University Medical Faculty, Izmir, Turkey; 3 Department of Public Health, Dokuz Eylul University Medical Faculty, Izmir, Turkey

**Keywords:** digoxin, therapeutic drug monitoring, heart failure, electronic health records

## Abstract

**Introduction:**

Measurement of serum digoxin concentrations before steady-state is reached results in a falsely low concentration, and may affect treatment safety. We evaluated the proportion of serum digoxin measurements performed before steady-state is reached and the reasons for inappropriate sampling in hospitalized patients.

**Materials and methods:**

Electronic medical records of patients hospitalized between January 2011 and December 2015 treated with oral digoxin, that had more than one digoxin measurement were included. Serum digoxin measurements performed before achievement of pharmacological steady state were considered as inappropriate. The chi-square and chi-square for trend tests were used to analyse the relationship between inappropriate measurements and age, gender, diagnosis, inpatient service, serum digoxin, potassium and creatinine concentrations.

**Results:**

We evaluated 2065 hospital admissions for 1621 patients and 11,407 digoxin measurements. The time between consecutive measurements was 1.9 ± 2.4 days and 97% of all measurements were classified as inappropriate. There was no releationship between patient age, gender, serum creatinine concentration and inappropriate measurement. As opposed to expected, inappropriate digoxin measurement was higher when potassium concentrations were within the normal range (P = 0.025). Share of inappropriate determinations of digoxin was higher when concentrations > 2.6 nmol/L were recorded (P < 0.05). These measurements were requested most often in coronary care unit and cardiology department.

**Conclusions:**

In our study, inappropriate serum digoxin measurement was found to be very high although only one of the appropriateness criteria was evaluated. The findings reveal the need for some strategies to prevent inappropriate measurements and reduce costs.

## Introduction

Although not as frequent as previously, digoxin is still being used in the management of chronic atrial fibrillation and mild to moderate heart failure (*1*, *2*). For effective and safe treatment with digoxin, therapeutic drug monitoring (TDM) should be performed because of its complex pharmacokinetic profile, narrow therapeutic index and extensive toxicity profile.

TDM is defined as the measurement of drug concentrations that, with appropriate clinical pharmacological interpretation, will directly affect prescribing procedures (*3*). Many factors contribute to an accurate and meaningful drug concentration measurement. One of them is correct sampling time. In general, blood should be drawn after steady state concentration is reached and just before the next dose (through concentration, 6-8 hours after drug intake). The studies showed that the time to reach steady state for digoxin is approximately 1 week (5 times the elimination half life) (*2*-*4*). Therefore, one should wait for at least 7 days before serum digoxin concentration is measured in cases when the treatment has just started or the dose has just been changed (*3*, *5*). Measurements performed with incorrect timing may produce misleading results which would lead to inappropriate change in clinical management of patients and waste economic resources (*3*, *4*).

While evaluating serum digoxin concentrations, besides blood sampling time, patients’ factors that alter the pharmacokinetics and pharmacodynamics of digoxin should be taken into consideration. Renal function is the major factor affecting serum digoxin concentrations (*2*-*8*). Metabolic abnormalities such as hypokalemia, hypomagnesemia, and hypercalcemia change tissue sensitivity to digoxin and toxicity may occur even within “therapeutic” serum concentrations (*2*, *7*). Also, medications used by the patient should be evaluated since drug interactions affect serum concentration of digoxin, usually through decreasing its renal clearance (*2*).

TDM for digoxin started nearly 50 years ago. However, inappropriate use of TDM for digoxin is still encountered despite so many years of practice. The present retrospective study was performed to assess the proportion of digoxin measurements in hospitalized patients not fulfilling accepted criteria for blood sampling in correct time and to identify reasons for inappropriateness.

## Materials and methods

### Study design

This study was designed as a descriptive, retrospective, electronic medical records database review at the Dokuz Eylul University Hospital. This study started after its approval by Dokuz Eylul University Ethics Committee for Non-Interventional Research and was performed in accordance with the Declaration of Helsinki.

The data of the patients hospitalized in Dokuz Eylul University Hospital between January 2011 and December 2015, treated with oral digoxin, and with more than one measurement of serum digoxin concentration, were included in this study. Each hospital admittance of a single patient was considered separately. Outpatients, patients who had only one measurement of serum digoxin concentration, and patients aged younger than 18 were excluded.

Electronic medical records were reviewed by the first author to obtain the following information: date, age, gender, diagnosis, digoxin result, hospital service, potassium concentration, creatinine concentration and time elapsed since preceding digoxin measurement.

### Appropriateness criteria for blood sampling

Correct sampling time for digoxin measurement is after steady state concentration is reached, which is approximately 7 days (*3*). Serum digoxin measurements made sooner than 7 days after the first measurement were considered as “inappropriate measurements”. Other appropriateness criteria is that blood should be sampled 6-8 hours after drug intake (through concentration). In our series, since blood collections were performed early in the morning before intake of drugs, we assume that samples were drawn at "through concentration".

### Methods

Serum digoxin concentrations were measured by particle-enhanced turbidimetric immunoassay (PETIA) using the Architect c8000 (Abbott Diagnostics Inc, Santa Clara, USA) between 2010 and 2013, by immuno-inhibition method with AU5800 (Beckman Coulter Inc, Brea, USA) between 2013 and 2015 in Dokuz Eylul University Central Laboratory. The therapeutic range of digoxin was defined as 1.0-2.6 nmol/L. Same trilevel internal quality control materials were used for both methods according to recommended values (Lyphochek Therapeutic Drug Monitoring Control, Cat no:450, Bio-Rad Laboratories Inc, Hercules, USA). The average coefficients of variation were 0.69% for Beckman Coulter assay and 0.66% for Abbott assay. The acceptance criteria for imprecision were < 0.25 total allowable error (TEa) (TEa for digoxin 20% according to CLIA Requirements for Analytical Quality).

### Statistical analysis

Data were presented as percentages, mean values with standard deviation or median with interquartile range (IQR). Categorical comparisons were performed using the chi-square and chi-square for trend test. A 2-sided P value of 0.05 was considered statistically significant. Data were analysed using SPSS 15.0 for Windows (SPSS Inc., Chicago, IL, USA).

## Results

Table 1 presents data of patients, including 2065 hospital admissions for 1621 patients and 11,407 serum digoxin measurements (after the first measurement).

**Table 1:** Characteristics of patients who had serum digoxin concentration measurements

**Age, years**	76 (18-109)
**Female, N (%)**	1094 (53)
**Diagnosis, %** atrial fibrillation heart failure atrial fibrilation and heart failure other *	11 43 14 32
Total hospitalisation, N	2065
SDC measurements, N^†^	11,407
Age is presented as median (range). * Other diagnoses by frequency: breathing abnormalities, heart beat abnormalities, chronic ischemic heart disease, respiratory failure, pneumonia. SDC - serum digoxin concentration. †Number of serum digoxin measurements after the first measurement.	

The time between consecutive serum digoxin concentration measurements was 1 day (IQR: 1-2 days). Almost all measurements were found to be performed within 7 days after the first measurement, which was defined as “inappropriate measurement” (Table 2).

**Table 2:** Time between consecutive serum digoxin concentration measurements

**The time between two consecutive measurements**	**SDC measurements,** N* (%)
Less than 7 days	11,033 (97)
1 day 2 days 3-6 days	7312 (64) 2008 (18) 1713 (15)
7 days and more	374 (3)
SDC - serum digoxin concentration. *Number of serum digoxin measurements after the first measurement.	

When we looked into the reasons for inappropriate measurements, no association was found between measurement frequency and patient age, gender, serum creatinine concentration (P > 0.05, data not shown). When serum digoxin concentration exceeded 2.6 nmol/L, which is the threshold value for toxic findings in most patients, the share of inappropriate measurements of digoxin was higher (Table 3). The measurements were inappropriate in 95%, 97% and 98% of patients with atrial fibrillation (AF), heart failure (HF) and AF+HF, consecutively (P < 0.001, data not shown).

**Table 3:** Frequency of inappropriate serum digoxin concentration measurements

**SDC,** **nmol/L (N)**	**Inappropriate** **measurements, %^†^**	P*
< 1.0 (3911)	96	**0.001**
1.0 - 2.6 (6744)	97	
> 2.6 (752)	98	
Total (11,407)	97	
SDC - serum digoxin concentration. *chi-square for trend test. N - number of serum digoxin measurements after the first measurement. ^†^Percent of rows.		

As opposed to expected, inappropriate serum digoxin measurement was higher when potassium concentrations were within the normal range (P = 0.025, data not shown).

Out of the inpatient services, those ordering the highest number of inappropriate measurements were coronary care unit and the cardiology service (Table 4).

**Table 4:** Inappropriate serum digoxin concentration measurement and time between consecutive measurements in various services

**Services (N)**	**Inappropriate** **measurements** **%^†^**	**The time in days between two consecutive measurements**
Coronary CU (1971)	99	1 (1-1)
Cardiology (5279)	98	1 (1-2)
ICU-Internal diseases (1102)	96	1 (1-2)
CVS (435)	94	1 (1-2)
Anesthesia ICU (488)	90	3 (3-4)
Other (2132)	93	1 (1-3)
	P* < 0.001	
Data are presented as median and interquartile range. ICU - Intensive Care Unit. Coronary CU - Coronary Care Unit. CVS - Cardio-Vascular Surgery. Other services - a total of 34 services including chest diseases, ICU of cardiothoracic surgery, orthopedics and traumatology, gastroenterology. *chi-square test. N - number of serum digoxin measurements after the first measurement. ^†^Percent of rows.		

## Discussion

Studies have shown that serum digoxin concentration measurements are not frequently performed according to the accepted criteria (*4*). This study showed that similar inappropriate measurements still take place in a university teaching hospital setting.

One of the main reasons for inappropriate measurement of digoxin is the measurement of serum digoxin concentrations before steady state concentration is reached. Interpreting a result before reaching steady state is quite difficult and may be wrong leading to inappropriate treatment decisions (*9*). In our study, 97% of the measurements were inappropriate. In other words, almost all measurements were made before waiting for the drug to reach steady state concentration. This ratio is quite high when compared to the results of the other studies (76%, 35% and 19%) (*7*, *8*).

Average number of digoxin measurements per patient during their stay at the hospital was 6.4 (range 2-91) (data not shown) and most of the measurements (82%) were performed at 1 to 2 day intervals. In fact, if the patients under digoxin treatment are stable both clinically and biochemically, the suggested interval to perform measurements extends as long as 10 months. It has been even stated that there is no need for routine follow up as long as there is no suspicion for theurapeutic insufficiency or toxicity, no medical condition to affect serum digoxin concentrations has emerged, or no change in drug consumption has been reported (*5*).

In our study as the serum concentration of digoxin increased, the inappropriate measurements increased. This finding can be explained by the doctors’ being highly concerned when the drug concentration is above therapeutic range. However, even in cases of digoxin toxicity such a frequent rate of measurement can not be justified (*3*, *6*). On the other hand, it is evident that inappropriate measurement ratio is still high (97%) even when serum concentration is within therapeutic range. The studies evaluating the reasons for inappropriate orders of laboratory tests showed that the influencing factors might be the anxiety of the doctors to make a mistake, their lack of experience, their lack of knowledge about appropriate use of the tests, their ordering the same test repeatedly before checking the result of the previous test, and the ease of access to laboratory tests (*9*). In our hospital, studies should be planned to find out the reasons for such high rates of inappropriate digoxin and possible other drug measurements and preventive action plans should be put into practice in accordance with the results.

Increased age, elevated serum creatinine concentration, and hypokalemia are among the reasons for increased risk for digoxin toxicity (*4*, *9*, *10*). In our study, there was no relationship between age, creatinine concentration and hypokalemia *vs* inappropriate measurement. Contrary to expectations, inappropriate measurement was higher when potassium concentration was within normal range. This makes us believe that digoxin measurements might have been ordered along with other daily routine blood tests performed during patient’s stay at the hospital.

Serum digoxin concentrations were measured at 1.5 day intervals in coronary care unit, and at 1.6 day intervals in cardiology service. Finding out that 65% of inappropriate measurements were ordered from cardiology service and coronary care unit invalidated our assumption that doctors in departments where digoxin is used more frequently are more knowledgeable about the pharmacokinetic properties of digoxin. Therefore, the doctors in cardiology clinics should be informed of this issue in the first place. Furthermore, an easily accesible consultation system with clinical pharmacology could be set in high volume clinics such as cardiology.

Since there is no TDM request form in our university, information was obtained from electronic patient database. Therefore, we did not know if the measurements were ordered with the proper indications or if the proper actions were taken after the results were obtained. The lack of evaluation on these appropriateness criteria is the limitation of the study.

In conclusion, we found that 97% of serum digoxin measurements performed during the hospital stay of patients were inappropriate, and that 65% of inappropriate measurements were ordered from cardiology service and coronary care unit.

We consider that the findings of the study are important since they draw attention to blood collection time in digoxin measurement and they reveal the need for a continuous medical education on TDM. TDM is a multidisciplinary team work including clinicians, nurses, biochemists and pharmacologists. Accurate blood concentrations can only be obtained by collaboration between the members of the team and their education on TDM. Another importance of the study is that it emphasizes the need for guidelines for serum drug concentration measurements and a TDM service. It also shows the need for upgrading electronic request forms to notify the user when the request is inappropriate. Increased awareness of appropriate use of TDM will prevent misleading laboratory test results, and will lead to considerable savings in health budget.
